# Long-term Treatment of Pediatric Metastatic Papillary Thyroid Cancer With Lenvatinib

**DOI:** 10.1210/jcemcr/luad175

**Published:** 2024-01-27

**Authors:** Julia R Donner, Bradley DeNardo, Lisa Swartz Topor

**Affiliations:** Department of Pediatrics, Hasbro Children's Hospital and the Warren Alpert Medical School of Brown University, Providence, RI 02903, USA; Division of Pediatric Hematology/Oncology, Hasbro Children's Hospital and the Warren Alpert Medical School of Brown University, Providence, RI 02903, USA; Division of Pediatric Endocrinology, Hasbro Children's Hospital and the Warren Alpert Medical School of Brown University, Providence, RI 02903, USA

**Keywords:** papillary thyroid cancer, radioactive iodine, tyrosine kinase inhibitors, lenvatinib, pediatric

## Abstract

Papillary thyroid carcinoma (PTC) is the most common pediatric thyroid malignancy and incidence is increasing. Standard treatment for PTC in pediatric patients includes surgical intervention, suppression of TSH with levothyroxine, and radioactive iodine therapy (RAI) in select patients. In the setting of metastatic PTC or PTC refractory to RAI therapy, tyrosine kinase inhibitors (TKIs), such as lenvatinib, may be used. Until recently, experience with these targeted agents were largely limited to adult patients with progressive or refractory PTC. More recently, increased experience with TKI therapy has been reported in the pediatric population, with case reports and small series describing short-term TKI use. We report the case of a 15-year-old girl with RAI-refractory metastatic PTC who achieved stable disease with long-term lenvatinib treatment for more than 5.5 years. Prospective, longitudinal studies of TKIs in RAI-refractory pediatric PTC are needed.

## Introduction

Thyroid cancer accounts for 1.5% to 3% of all childhood cancers, though the incidence has been increasing (1). Papillary thyroid cancer (PTC) is the predominant pediatric thyroid malignancy and is the most common secondary malignancy in survivors of childhood cancer. Gene rearrangements of the *RET* oncogene are the most common molecular alteration in pediatric PTC, with rearrangements of *NTRK* and point mutations in *BRAF* occurring less frequently (2). Treatment for pediatric metastatic PTC includes thyroidectomy and lymph node dissection, with adjunctive radioactive iodine (RAI) therapy and thyroid hormone replacement with levothyroxine to suppress TSH (1, 2).

Historically, patients with progressive metastatic PTC no longer amenable to surgery or RAI have had limited systemic treatment options because cytotoxic chemotherapy agents lack long-term efficacy (2). The emergence of oral multitargeted tyrosine kinase inhibitors (TKIs) that inhibit oncogenic intracellular signaling pathways or exert antiangiogenic effects has dramatically improved treatment options for such patients; somatic mutation testing is necessary to identify targetable alterations if present. Lenvatinib is a multitargeted TKI that inhibits numerous signaling pathways including RET, VEGF, and FGFR (2) and was approved by the Food and Drug Administration in 2015 for the treatment of RAI-refractory differentiated thyroid cancer (DTC) in adults. A randomized phase III clinical trial of lenvatinib vs placebo demonstrated significantly improved progression-free survival in patients with RAI-refractory DTC who were aged >18 years (3). There are few reported cases of lenvatinib use in pediatric PTC and long-term treatment is not described. Here, we describe the long-term use of lenvatinib in a pediatric patient with RAI-refractory PTC.

## Case Presentation

**Figure 1.**
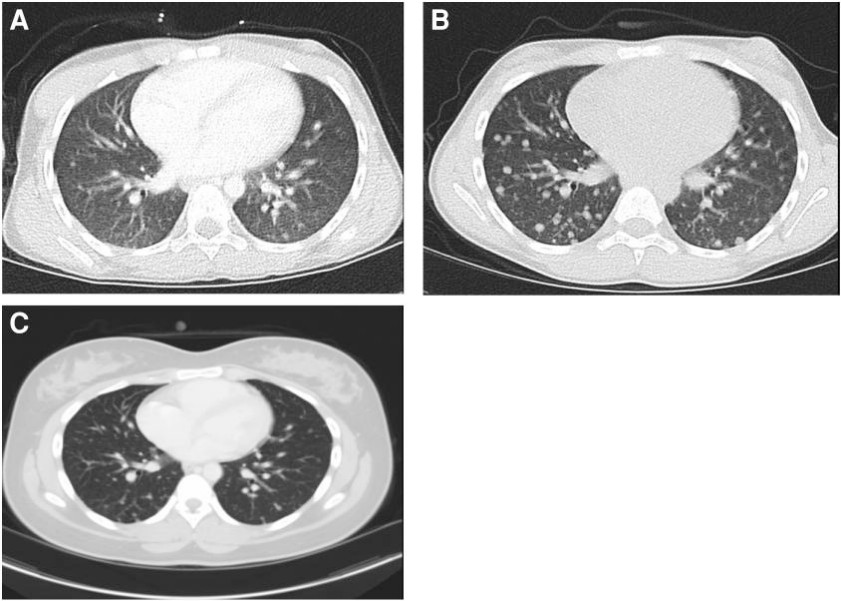
Computed tomography (CT) of the chest. (A). January 2016: initial CT of the chest demonstrating diffuse pulmonary nodules. (B). May 2017: follow-up CT of the chest with increased size and number of pulmonary nodules 6 months after second RAI treatment. (C). September 2023: follow-up CT of the chest with stable/unchanged pulmonary nodules.

In September 2015, a 7-year-old girl who had recently immigrated to the United States presented with a firm mass replacing the entire thyroid and bilateral anterior neck. Cytology obtained by fine needle aspiration was consistent with malignancy, and she underwent a planned 2-stage total thyroidectomy with bilateral neck dissection in the 3 months following initial diagnosis. Pathological examination showed PTC, diffuse sclerosing variant. Multiple bilateral lymph nodes were involved by metastatic PTC, with extracapsular extension, extensive involvement of the vascular space, and tumor wrapped around the recurrent laryngeal nerve. Molecular analysis of the tumor identified a somatic RET-PTC3 fusion. Two weeks after the second surgery, a neck ultrasound showed a slight interval increase in the size of lesions within the left thyroid bed, suggestive of residual tumor. Diagnostic whole-body scan with RAI I-131 revealed metastatic disease with several foci of abnormal iodine uptake in the neck, left supraclavicular region, and bilateral lungs diffusely. She received therapeutic RAI at a reduced dose of 52 mCi to minimize risk of pulmonary fibrosis, and posttreatment tumor uptake was confirmed on whole body I-131 scan 7 days later. Subsequent computed tomography (CT) of the neck and chest with IV contrast 1 month after RAI showed residual enhancing tissue in the thyroid bed and neck as well as innumerable pulmonary nodules between 2 and 6 mm in size (Fig. 1A).

## Diagnostic Assessment

One year following RAI therapy, disease progression was identified on follow-up imaging. CT of the neck and chest revealed disease progression in the neck and lungs. She was treated with repeat neck dissection followed by a second course of RAI therapy at a dose of 125 mCi. Additionally, the patient required a tracheostomy because of recurrent episodes of stridor resulting from laryngeal cord dysfunction after surgery. Six months after the second RAI therapy, repeat CT of the neck and chest revealed further disease progression with an interval increase in the size and number of pulmonary nodules, the largest of which measured 12 mm. Following 5 additional months of surveillance, CT showed continued pulmonary nodule growth (Fig. 1B) and the decision was made to initiate TKI therapy with lenvatinib in January 2018.

## Treatment

Lenvatinib at a dose of 14 mg (12.6 mg/m^2^) by mouth once daily was initiated 27 months after initial diagnosis. Eleven months after initiation of lenvatinib, the patient experienced dermatologic toxicity with skin peeling, blistering of the soles of the feet, and significant pain consistent with hand-foot syndrome (HFS) prompting dose modification (4). Lenvatinib was held for 2 weeks with improvement of skin symptoms and then was restarted at a dose of 10 mg daily. Approximately 4.5 months later, foot pain returned, and the lenvatinib was held for 2 weeks. She restarted at a final dose of 4 mg once daily and has not experienced any further symptoms of recurrent HFS. The patient has experienced no other toxicities or laboratory abnormalities attributable to lenvatinib.

## Outcome and Follow-up

**Figure 2.**
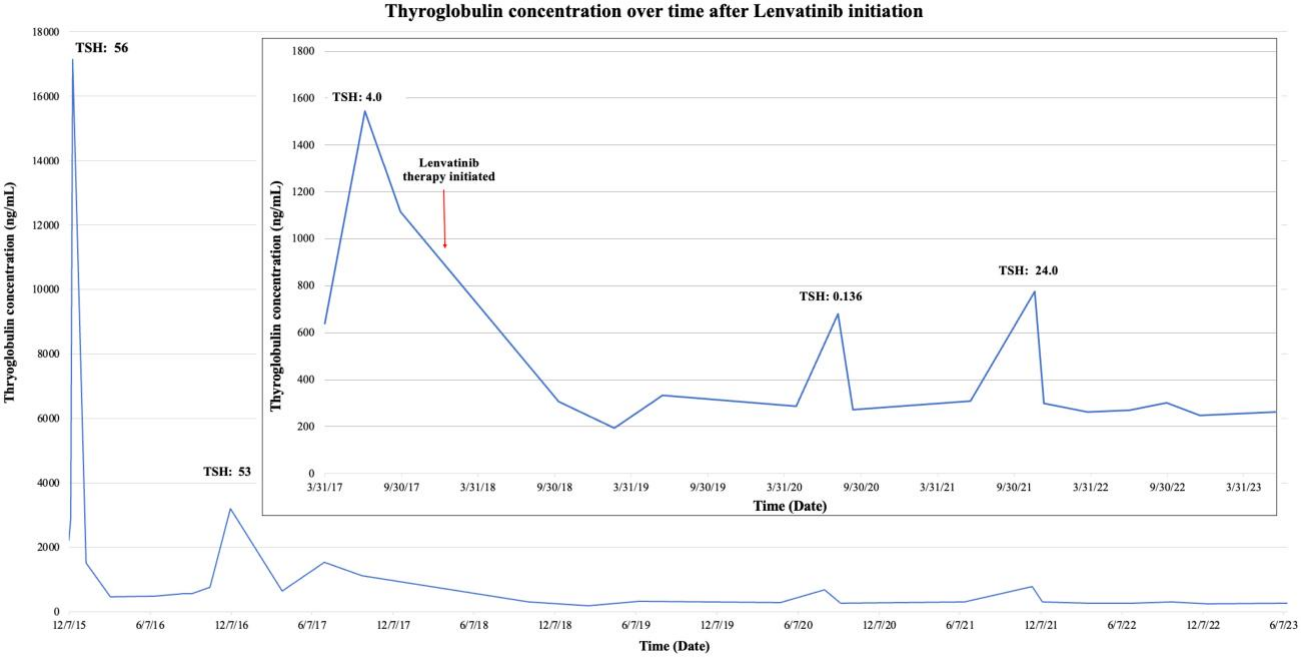
Serum thyroglobulin concentration over time.

The patient has remained on lenvatinib 4 mg once daily for 5.5 years with excellent compliance, and treatment is ongoing. Rise in thyroglobulin levels did occur in conjunction with periods of noncompliance with levothyroxine, with a decrease in thyroglobulin levels after TSH returned to target range with improved compliance (Fig. 2). With long-term lenvatinib therapy, the patient has had no evidence for disease progression on serial disease evaluations consisting of a CT of the neck and chest, neck ultrasound, and thyroglobulin level tests every 3 to 6 months. Most recent imaging demonstrates no disease recurrence within the neck and unchanged bilateral pulmonary nodules (Fig. 1C). Additionally, she has had resolution of prior respiratory symptoms with decannulation of tracheostomy 4.5 years after initiating TKI therapy. Routine follow-up with pediatric endocrinology and oncology has revealed no evidence for late effects from chronic lenvatinib treatment.

## Discussion

Refractory PTC is challenging to treat in children because of limited therapeutic options. Small molecule TKIs are approved for the treatment of RAI-refractory DTC in adults. A randomized, double-blind, phase III clinical trial of lenvatinib in adults demonstrated a 14.7-month increase in median progression-free survival compared with placebo (5). Several case reports in pediatric patients with RAI-refractory PTC demonstrate the potential benefit of short-term TKI therapy, although long-term follow-up is not well described and clinical trials are ongoing. Majahan and colleagues described 3 pediatric patients with metastatic PTC and pulmonary disease treated with lenvatinib. All 3 patients were able to wean off supplemental oxygen therapy following lenvatinib initiation. Two of the patients were treated with lenvatinib for a maximum duration of 23 months, achieving stable disease and improved quality of life (6). Dujovne and colleagues described a 10-year-old girl with metastatic PTC reliant on supplemental oxygen due to diffuse pulmonary disease for whom total thyroidectomy and lymph node dissection was not possible because of extensive local infiltration. She was started on lenvatinib through compassionate use and was able to wean off supplemental oxygen and leave the hospital within 9 days of treatment initiation; her disease burden was stable over 4 months before proceeding with surgery (7). To the best of our knowledge, this case represents the longest reported duration of lenvatinib therapy in a pediatric patient with refractory PTC who achieved stable disease burden and symptom improvement with ongoing therapy at more than 5.5 years.

Although TKI therapy with agents including lenvatinib and sorafenib have demonstrated favorable clinical outcomes in clinical trials, they have broad toxicity profiles, and dose modifications are common (8). Common adverse effects of lenvatinib include hypertension, diarrhea, skin/hair/mucous membrane alterations including HFS, fatigue, decreased appetite, weight loss, and nausea (5). In clinical trials in adult patients with advanced RAI-refractory DTC, grade 3 or higher toxicities were reported in 75% of patients taking lenvatinib (9). Most adverse effects developed within the first month of treatment and required dose reduction (8). Optimal duration of therapy and long-term toxicity of these therapeutic agents are not well defined. Among adult patients with RAI-refractory DTC treated for a median of 39.96 months of lenvatinib, 80% developed a new side effect after 12 months of therapy. Cardiovascular toxicity was most common, including arterial hypertension and atrial fibrillation. Diarrhea, proteinuria, thrombosis, and pulmonary embolism were also identified as late effects. The median age of the cohort was 66 years (10). The long-term side effects of lenvatinib treatment in children and optimal length of therapy in pediatric PTC are unknown. After 5.5 years of therapy, our patient has developed no appreciable late toxicity and early HFS has not recurred.

Over the past 5 years, more TKIs have become available. Although lenvatinib, a multikinase inhibitor with nonselective RET inhibition was the best option for treatment initiation in 2018, specific TKIs that are selective RET inhibitors, such as selpercatinib and pralsetinib, are now options for treatment (5). Our patient will likely transition to one of these in the future, especially if there is evidence of tumor growth. In addition, she may benefit from treatment that allows redifferentiation of the tumors, so RAI therapy may be an option in the future (11).

This case describes long-term lenvatinib treatment over 5.5 years that has been effective and well-tolerated in a case of childhood RAI-refractory metastatic PTC harboring RET-PTC3 fusion. Chronic lenvatinib therapy in our patient has resulted in prolonged stabilization of disease based on serial neck and chest CT images and thyroglobulin levels. Clinical trials of targeted therapy with oral TKIs such as lenvatinib are needed in pediatric patients with RAI-refractory DTC to define the efficacy and appropriate doses of these agents. When treating pediatric patients with RAI-refractory PTC with TKIs, questions regarding optimal duration of therapy and risks of late toxicity are unanswered. Treatment must be individualized on a case-by-case basis.

## Learning Points

- Surgery, TSH suppression with levothyroxine, and RAI are the first-line therapies for pediatric papillary thyroid carcinoma but may not always be effective.
- Tyrosine kinase inhibitors in pediatric patients can be used as an adjunct treatment in RAI-refractory papillary thyroid carcinoma.
- Tyrosine kinase inhibitors carry risks of treatment-related adverse events that may require dose reductions or therapy adjustments.

## Contributors

All authors made individual contributions to authorship. L.S.T. and B.D. contributed to the clinical care of the patient and J.R.D., B.D., and L.S.T. were involved in drafting and revising the manuscript. All authors reviewed and approved the final draft.

## Data Availability

Data sharing is not applicable to this article as no datasets were generated or analyzed during the current study.

## Abbreviations

CT: computed tomography
DTC: differentiated thyroid cancer
HFS: hand-foot syndrome
PTC: papillary thyroid cancer
RAI: radioactive iodine therapy
TKI: tyrosine kinase inhibitor
